# Exome Array Analysis of Susceptibility to Pneumococcal Meningitis

**DOI:** 10.1038/srep29351

**Published:** 2016-07-08

**Authors:** Anne T. Kloek, Jessica van Setten, Arie van der Ende, Michiel L. Bots, Folkert W. Asselbergs, Mercedes Valls Serón, Matthijs C. Brouwer, Diederik van de Beek, Bart Ferwerda

**Affiliations:** 1 Department of Neurology, Center of Infection and Immunity Amsterdam (CINIMA), Academic Medical Center, Amsterdam, the Netherlands; 2 Department of Cardiology, Division Heart & Lungs, University Medical Center Utrecht, Utrecht, The Netherlands; 3 Department of Medical Microbiology, The Netherlands Reference Laboratory for Bacterial, Meningitis, Center of Infection and Immunity Amsterdam (CINIMA), Academic Medical Center, Amsterdam, the Netherlands; 4 Julius Center for Health Sciences and Primary Care, University Medical Center Utrecht, Utrecht, The Netherlands; 5 Durrer Center for Cardiogenetic Research, ICIN-Netherlands Heart Institute, Utrecht, the Netherlands; 6 Institute of Cardiovascular Science, Faculty of Population Health Sciences, University College London, London, United Kingdom

## Abstract

Host genetic variability may contribute to susceptibility of bacterial meningitis, but which genes contribute to the susceptibility to this complex disease remains undefined. We performed a genetic association study in 469 community-acquired pneumococcal meningitis cases and 2072 population-based controls from the Utrecht Health Project in order to find genetic variants associated with pneumococcal meningitis susceptibility. A HumanExome BeadChip was used to genotype 102,097 SNPs in the collected DNA samples. Associations were tested with the Fisher exact test. None of the genetic variants tested reached Bonferroni corrected significance (p-value <5 × 10^−7^). Our strongest signals associated with susceptibility to pneumococcal meningitis were rs139064549 on chromosome 1 in the *COL11A1* gene (p = 1.51 × 10^−6^; G allele OR 3.21 [95% CI 2.05–5.02]) and rs9309464 in the *EXOC6B* gene on chromosome 2 (p = 6.01 × 10^−5^; G allele OR 0.66 [95% CI 0.54–0.81]). The sequence kernel association test (SKAT) tests for associations between multiple variants in a gene region and pneumococcal meningitis susceptibility yielded one significant associated gene namely *COL11A1* (p = 1.03 × 10^−7^). Replication studies are needed to validate these results. If replicated, the functionality of these genetic variations should be further studied to identify by which means they influence the pathophysiology of pneumococcal meningitis.

Acute bacterial meningitis is a life-threatening disease associated with substantial morbidity and mortality and ranks among the top 10 infectious causes of death^1^,^2^. *Streptococcus pneumoniae* is the leading causative pathogen and is identified in 70% of adult patients^3^,^4^. Despite early antibiotic and anti-inflammatory therapy, case fatality rates of pneumococcal meningitis range from 18–30%, and neurological sequelae, including hearing loss, focal neurological deficits, and cognitive impairment, occur in 30–55% of surviving patients^4^,^5^,^6^,^7^,^8^,^9^,^10^.

Several factors have been identified that contribute to the susceptibility to pneumococcal meningitis^11^. For example, patients with an immunocompromised state, organ transplantation, splenectomy or anatomical defects with cerebrospinal fluid leakage are at increased risk of pneumococcal meningitis^12^,^13^,^14^,^15^. Genetic variation leading to difference in immune response has also been identified to influence susceptibility to pneumococcal meningitis. Extreme phenotype studies looking at recurrent pneumococcal disease have shown that genetic variation in complement factor 2 (*C2*), interleukin-1 receptor-associated kinase 4 (*IRAK4*) and nuclear factor κB essential modulator protein (*NEMO*) were the cause of this increased susceptibility^16^. Furthermore, case-control studies mostly looking at variants in genes involved in the innate immune system have pointed out genetic variants associated with acquiring invasive pneumococcal disease^16^,^17^,^18^,^19^,^20^. In a Dutch case-control study genetic variations in the beta2-adrenoreceptor (*ADRB2*) and mannose binding lectin (*MBL*) genes were associated with susceptibility to pneumococcal meningitis^17^,^18^. These genetic association studies evaluated one or only few single-nucleotide polymorphisms within one gene in relatively small cohorts. A hypothesis-free approach in larger groups of patients and controls is needed to unravel new genetic risk factors for the susceptibility to pneumococcal meningitis. The aim of our study was to identify new genetic variants involved in susceptibility to pneumococcal meningitis, genotyping over 240,000 genetic variants of which the majority is exonic or otherwise functional.

## Results

Between 2006 and 2011, 656 patients with bacterial meningitis were included in the MeninGene study^21^. Of these patients, 469 (72%) had pneumococcal meningitis. After quality control filters 408 pneumococcal meningitis patients and 2072 controls were included in the genetic associations analysis for meningitis disease susceptibility. Demographic and clinical data of the successfully genotyped patients can be found in Table 1. After exclusion of all monomorf and sex chromosome variants a total of 100,464 single nucleotide polymorphisms (SNPs) passed quality control thresholds of >95% call rate and Hardy Weinberg equilibrium and were incorporated in the association analysis.

The genomic control parameter **λ** was 0.31 when all variations were included. This was driven by the rare variants and exclusion of variants with a minor allele frequency below 0.01%, increased λ to 0.93 (Supplementary Fig. 1). For our single marker analysis we therefore included those with a minor allele frequency higher than 0.01%.

**Figure 1:**
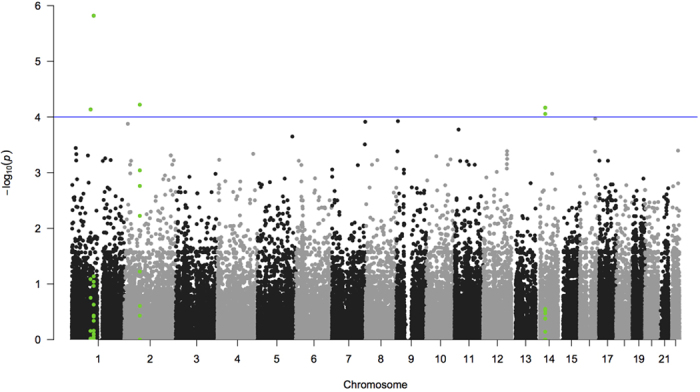
Manhattan plot of p-values. The y-axis indicates the –log_10_ (p*-*values) of the SNPs in the association analysis and the x-axis indicates the chromosomal position. The horizontal blue line indicates the threshold of p = 1 × 10^−4^. All markers of the three genes with the highest association signal, namely *COL11A1* (rs139064549, p-value = 1.51 × 10^−6^, location chromosome 1), *EXOC6B* (rs9309464, p-value = 6.01 × 10^−5^, location chromosome 2) and *FSCB* (with rs3809429, rs3825630 rs1959379, p-value = 6.80 × 10^−5^, 6.80 × 10^−5^, 8.81 × 10^−5^ location chromosome 14), have been colored green.

None of the tested genetic variants reached the Bonferroni corrected significance threshold (p-value <5 × 10^−7^). Six genetic variants associated with pneumococcal meningitis reached p-values lower than 1 × 10^−4^ (Fig. 1). Our strongest signal was the missense rs139064549 in the collagen type XI alpha 1 (*COL11A1*) gene (p = 1.51 × 10^−6^; G allele OR 3.21 [95% CI 2.05–5.02]) and the second strongest was the intron variant rs9309464 in the exocyst complex component 6B (*EXOC6B*) gene (p = 6.01 × 10^−5^; G allele OR 0.66 [95% CI 0.54–0.81]; Table 2). Of these six variants with a p-value lower than 1 × 10^−4^, three variants were located in the fibrous sheath CABYR binding protein (*FSCB*) gene, namely rs3809429 (p = 6.80 × 10^−5^; A-allele OR 1.65 [95% CI 1.30–2.09]), rs3825630 (p = 6.80 × 10^−5^; G allele OR 1.65 [95% CI 1.30–2.09]), and rs1959379 (p = 8.81 × 10^−5^; A allele OR 1.64 [95% CI 1.30–2.09]). The sixth variant rs617169 (p = 7.33 × 10^−5^; G allele OR 0.72 [95% CI 0.61–0.85]) was not located within a gene, but the nearest gene was protein kinase N2 (*PKN2)* located on chromosome 2.

In the single marker analysis we did not include variants with a MAF below 0.01%. To assess the role of these rare variants in the association testing of pneumococcal susceptibility we used the region based sequence kernel association test (SKAT) allowing us to include all common and rare variations within a gene region. By using the SKAT analysis, we found one significant associated gene namely *COL11A1* (p = 1.03 × 10^−7^). In this analysis we tested a total of 12968 gene sets and after correcting for these multiple tests *COL11A1* reaches a significance of p = 0.001. No difference in the p-value of *COL11A1* was observed when correcting for the proportion of case-control imbalance. The second strongest signal in the SKAT analysis was the polymerase (DNA directed) lambda (*POLL*) gene (p = 8.10 × 10^−5^), which was not significant after multiple testing correction. When corrected this p-value for proportion of case-control imbalance the association was slightly more significant (p = 7.05 × 10^−5^). We also looked if the portion of common and rare variants drove the associations with pneumococcal meningitis susceptibility when the rare variants threshold was defined as a function of the total sample size^22^. This analysis showed only for *POLL* (p = 1.90 × 10^−5^) that association was based on the proportion of common and rare variants, although this did not reach genome-wide significance after correction for multiple testing.

## Discussion

We performed the first hypothesis free exome-wide association study on disease susceptibility in a large homogeneous group of pneumococcal meningitis patients. We did not identify a significant association in our single marker analysis after correction for multiple testing. The strongest associations of single nucleotide variations with pneumococcal meningitis susceptibility are found within genes that have not been related to pneumococcal meningitis or pneumococcal disease previously. Our strongest signal (rs139064549) is located in the *COL11A1* gene, which encodes for one component of type XI collagen that adds structure and strength to connective tissues. *COL11A1* has a fundamental role in the control of fibrillogenesis and mutations in this gene are associated with Stickler syndrome^23^,^24^. Stickler syndrome is a genetic disorders affecting connective tissue and is characterized by distinctive facial abnormalities, ocular problems, hearing loss and joint problems^24^. Related gene ontologies of the *COL11A1* gene are extracellular matrix structural constituents and extracellular binding. The association of the *COL11A1* gene could indicate that differences in the extracellular matrix or anatomical defects can alter the susceptibility of pneumococcal meningitis. The second strongest association (rs9309464) is located in an intronic region of the *EXOC6B* gene. The *EXOC6B* gene is part of a multiprotein complex required for targeted exocytosis. This complex is highly expressed in the central nervous system and is important for cell polarity, growth and neuronal cell migration^25^. In previous studies, genetic variations affecting the *EXOC6B* gene have been associated with cerebral disorders, like intellectual disability, but associations with infectious diseases have not been reported^26^. The three genetic associations found in the *FSCB* gene are all intron variants. FSCB is a testis specific protein and in mice it is suggested be involved in the later stages of fibrous sheath assembly of spermiogenesis^27^. The *FSCB* gene is not described to be associated with neurological or infectious diseases in earlier studies. Since rs617169, the fifth strongest associated variant is located outside any gene and therefore we could not explain the possible involvement during pneumococcal meningitis susceptibility. The closest gene to rs617169 is the *PKN2* gene encoding for a multifunctional protein kinase N2^28^.

The SKAT analysis, performed to identify differences between patients and controls in the prevalence of a set of variants within genes, showed the association of one gene; *COL11A1*. As expected, most rare variants were found in the largest group, namely the control group. This finding suggests that the fivefold difference in amount of patients and controls is responsible for the significant differences we have found. We tried to determine this effect by applying the hybrid SKAT resampling method to test the proportion of case-control imbalance. This did not result in a noteworthy change of the p-value. Consequently, this indicates that a larger number of patients is needed to determine the true role of rare variants in pneumococcal meningitis susceptibility. As described above, we found a marginally significant association of the *COL11A1* gene with pneumococcal meningitis susceptibility. The second highest association is found in the *POLL* gene and was not significance after correction for multiple testing*. POLL* encodes for a DNA polymerase, which participates in V(D)J recombination to generate B-cell and T-cell receptor diversity. In animal models *POLL* seems to be important for light-chain rearrangements^29^. The potential role of *POLL* in B-cell and T-cell differentiation could explain that genetic variation in this gene influences pneumococcal meningitis susceptibility.

Previous genetic association studies in pneumococcal meningitis susceptibility have used a hypothesis driven approach in relatively small cohorts^16^,^17^,^18^,^19^,^20^,^30^. For example, association of a set of polymorphisms in the *MBL2* gene resulted in a p-value of 0.017 (OR 8.21 [95% CI 1.05–64.09]) and the highest association of a SNP in the *ADRB2* gene had a p*-*value of 0.007 (OR 1.52 [95% CI 1.12–2.07]) were reported. In our data the set of SNPs in *MBL2* gene reached a p-value of 0.09 (OR 1.65 [95% CI 0.91–2.98]) and the same genetic variation in the *ADRB2* gene a p-value of 0.164 (OR 1.12 [95% CI 0.1–1.3]). The associations found in those studies were not very strong and both studies concluded validation in larger populations is needed^17^,^18^. The challenge in genetic association studies looking at infectious diseases is the reproducibility of the data. The strength of our unique pneumococcal meningitis patient cohort is the homogenous group of patients but we are limited in replication of our findings in a similar group of patients. Especially since we found one gene with exome wide significance, replication of this association within another patient population is essential to validate this result.

Another limitation of our study is that by using the exome SNP array, it remains unclear if the associated genetic variants are also the causal variants with a biological function. Potentially the identified variants are in linkage disequilibrium -pair wise correlation between nearby genetic variants- with the causal variants.

We observed minimal deviation from the null for variants with MAF > 0.2 with a p-value < 0.01 (see supplement Fig. 1A). Permutation-based approach did increase lambda to 1.02, but did not affect the early deviation from the null. Likely causes of the early deviation are the technical (genotyping) and population differences between the cases and controls, or missing covariates such as antibiotic use or occurrence of co-infections (supplement Fig. 1C).

Our current study did not detect previously reported associations. This could be explained by our homogenous category of pneumococcal meningitis patients. Susceptibility to invasive pneumococcal infections is strongly dependent on host-pathogen interaction, therefore specifying the causing pathogen and type of infection can be crucial in determining specific risk genes for a complex trait like pneumococcal meningitis.

## Conclusion

Our study reveals new genetic variants associated with the susceptibility to pneumococcal meningitis. These genetic variations were just below exome-wide significance. We found one gene significantly associated when looking for associations between multiple common and rare variants with pneumococcal meningitis susceptibility, namely the *COL11A1* gene. These findings need to be replicated in an independent pneumococcal meningitis patient cohort. Following replication studies, the role of these genes in the pathogenesis of pneumococcal meningitis needs to be determined. Increasing our biological insight into the pathogenesis of bacterial meningitis brings us one step closer to understanding the disease, and discovering new therapeutic and preventive strategies.

## Methods

### Study cohort and data collection

Patients included in this study are part of a prospective nationwide observational cohort study in the Netherlands; the MeninGene study^4^. For this study, all patients of 16 years and older with a community acquired bacterial meningitis were included from 2006 to 2011. Bacterial meningitis was defined by positive culture of a bacterial pathogen in the cerebrospinal fluid or a positive polymerase chain reaction test for a bacterial pathogen in the cerebrospinal fluid in combination with specific cerebrospinal fluid findings indicative of bacterial meningitis^31^. The Netherlands Reference Laboratories for Bacterial Meningitis (NRLBM) provided researchers of the MeninGene study with a daily update of where patients with bacterial meningitis have been admitted in the preceding days. Furthermore, physicians could contact the researchers to include patients with a 24/7 telephone service. Patients with hospital acquired bacterial meningitis, severe head trauma or neurosurgery in the previous month, or with a neurosurgical device in the central nervous system were excluded. Online case-record forms were used to collect data on patients’ history, symptoms and laboratory findings during admission, treatment, clinical course during hospitalized period and outcome at discharge.

Blood for DNA extraction was drawn from the patients and collected in sodium/EDTA tubes. Isolation of the DNA was performed with the Gentra Puregene isolation kit (Qiagen) according to manufacturer’s protocol. Thereafter the yield and quality of the extractions were determined to ensure appropriate conditions for genotyping.

The control population comprised 2072 individuals from the Utrecht Health project^32^. The Utrecht Health Project (UHP) is an ongoing dynamic population study initiated in a newly developed large residential area in Leidsche Rijn, part of the city of Utrecht. All new inhabitants were invited by their general practitioner to participate in the UHP. Of all participants an individual health profile (IHP) was made by dedicated research nurses. In a sample of the UHP participants genomic data has been collected.

### Genotyping and quality control

All bacterial meningitis patients and controls were genotyped using the Illumina Exome array v1.1 in collaboration with the Human Genome Facility and the department of Epidemiology, Erasmus MC, the Netherlands as part of the Netherlands ExomeChip Project. After genotyping we performed calling on our MeninGene samples and to improve calling accuracy samples were joined with UHP and other Utrecht BBMRI-NL cohorts comprising N = 9844 samples. The samples from the other Utrecht BBMRI-NL cohorts were excluded after quality control (QC). All autosomal SNPs were called using GenomeStudio software, from Illumina, using the GenTrain 2.0 cluster algorithm using the protocol as described in Guo *et al*.^33^.

Called data was exported in PLINK format for final sample and SNP QC^34^. High quality, independent SNPs with less than 1% missing values, no significant differences in missing values between cases and controls, minor allele frequency (MAF) > 5% and Hardy-Weinberg equilibrium (HWE) > 1 × 10^−3^ were used to determine relatedness, heterozygostiy and ancestry. Individuals with a minimal second degree of relatedness, or higher degree relatedness and heterozygosity above or below three standard deviations were left out. To avoid population stratification we used multidimensional scaling (MDS), implemented in PLINK, on all meningitis cases, controls and HapMap samples to select only those patients that clustered with the European CEU HapMap samples (Supplementary Fig. 2)^35^. Finally we performed QC by removing all SNPs that had >5% missing values and HWE p-value <1 × 10^−6^.

### Statistical analyses

Differences between cases and controls were calculated using the Fisher’s exact test on allelic association using PLINK 1.9^36^. To correct for multiple testing we applied the Bonferroni correction (P < 0.05/number of analyzed SNPs). To investigate if permutation-adjustment removed the inflation permutation adjusted p-values after 1,000,000 permutations have been calculated using PLINK. Because we excluded all the variants with a frequency below 1% we also wanted to test if there was a significant association between pneumococcal meningitis patients and healthy controls with the inclusion of these rare variants. To test this we applied the gene-based test Sequence Kernel Association Test (SKAT) adaptive sum to test the association of the phenotype and SNP sets per gene with all variations having the same weight^22^,^37^. Secondly we tested all genes with the combined test of rare and common variants to look at the effect of rare variants^22^,^37^. For the gene-based SKAT test we corrected for any population stratification by including the first four MDS dimensions. To determine the proportion of case-control imbalance we used implemented hybrid-resampling method built in SKAT^22^,^37^.

### Ethics statement

The MeninGene study was approved by the ethics committee of the Academic Medical Center, Amsterdam, The Netherlands. Ethical approval was granted in all participating centers and written informed consent was obtained from all participating individuals or legally authorized representatives. The study was conducted according to the principles of the Declaration of Helsinki (version of 2013, Fortaleza, Brazil) and in accordance with the Medical Research Involving Human Subjects Act (WMO) and other guidelines, regulations and acts. The UHP study was approved by the Medical Ethical Committee of the University Medical Center, Utrecht, The Netherlands and written informed consent was obtained from all participants.

## Additional Information

**How to cite this article**: Kloek, A. T. *et al*. Exome Array Analysis of Susceptibility to Pneumococcal Meningitis. *Sci. Rep.* **6**, 29351; doi: 10.1038/srep29351 (2016).

## Supplementary Material

Supplementary Information

## Figures and Tables

**Table 1:** Demographic and clinical data of the included pneumococcal meningitis patients.

Characteristics	Pneumococcal meningitis patients (n = 408)
Number of male cases (%)	190/408 (47%)
Mean age, years (SD)	59 yrs. (14)
Symptoms before admission:	
Seizures (%)	29/389 (8%)
Duration of symptoms <24 hours (%)	199/391 (51%)
Predisposing conditions	
Sinusitis/otitis media (%)	189/401 (47%)
Pneumonia (%)	40/390 (10%)
Immunocompromised state (%)	103/402 (26%)
Clinical characteristics on admission	
Classic triad (%)	208/391 (53%)
Coma (%)	57/402 (14%)
Laboratory characteristics on admission	
Median CSF leukocyte count (IQR)	4500 cells/3mm^3^ (941–10856)
Median CRP (IQR)	194 mg/L (83–306)
Mean thrombocyte count (SD)	218 × 10^9/L (86)
Unfavorable outcome (%)	135/402 (34%)
Death (%)	31/402 (8%)

**Table 2:** Susceptibility to pneumococcal meningitis (6 lowest P-values/Fisher exact test).

Chr	Basepair	Rs number	Gene	Alleles		A1 allele frequency		P-value		OR (+/− 95 CI)
				A1	A2	Cases	Control	Unadjusted	BONF	
1	103354135	rs139064549	*COL11A1*	G	C	0.0393	0.0126	1.51 × 10^−6^	0.15	3.21 (2.05–5.02)
2	72533963	rs9309464	*EXOC6B*	G	A	0.146	0.206	6.01 × 10^−5^	1	0.66 (0.54–0.81)
14	44975606	rs3809429	*FSCB*	G	A	0.123	0.0784	6.80 × 10^−5^	1	1.65 (1.30–2.09)
14	44975052	rs3825630	*FSCB*	A	C	0.123	0.784	6.80 × 10^−5^	1	1.65 (1.30–2.09)
1	88745673	rs617169	–	G	A	0.281	0.353	7.33 × 10^−5^	1	0.72 (0.61–0.85)
14	44974966	rs1959379	*FSCB*	A	G	0.123	0.0785	8.81 × 10^−5^	1	1.64 (1.30–2.09)
