# Supplementary material for: Exome Array Analysis of Susceptibility to Pneumococcal Meningitis
Source: Sci Rep. 2016 Jul 8;6:29351. doi: 10.1038/srep29351 (PMC4937363; doi:10.1038/srep29351)

EXOME ARRAY ANALYSIS OF SUSCEPTIBILITY TO PNEUMOCOCCAL MENINGITIS

Anne T. Kloek[1], Jessica van Setten[2], Arie van der Ende[3], Michiel L. Bots[4], Folkert W. Asselbergs[2,5,6], Mercedes Valls Serón [1], Matthijs C. Brouwer[1], Diederik van de Beek[1†*], Bart Ferwerda[1†]

1. Department of Neurology, Center of Infection and Immunity Amsterdam (CINIMA), Academic Medical Center, Amsterdam, the Netherlands.
2. Department of Cardiology, Division Heart & Lungs, University Medical Center Utrecht, Utrecht, The Netherlands.
3. Department of Medical Microbiology, The Netherlands Reference Laboratory for Bacterial Meningitis, Center of Infection and Immunity Amsterdam (CINIMA), Academic Medical Center, Amsterdam, the Netherlands.
4. Julius Center for Health Sciences and Primary Care, University Medical Center Utrecht, Utrecht, The Netherlands.
5. Durrer Center for Cardiogenetic Research, ICIN-Netherlands Heart Institute, Utrecht, the Netherlands;
6. Institute of Cardiovascular Science, Faculty of Population Health Sciences, University College London, London, United Kingdom.

[†] These authors contributed equally to this work

* Corresponding author

Address correspondence:

Diederik van de Beek

Department of Neurology

Academic Medical Center, University of Amsterdam

PO Box 22660

1100DD Amsterdam, The Netherlands

Telephone +31205663647

Fax +31205669374

E-mail: d.vandebeek@amc.nl

SUPPLEMENTARY INFORMATION/FIGURES

Supplementary figure 1: Quantile quantile plot of p-values with MAF >1%. The X-axis indicates the expected $-\log_{10}$ (p-values) and the y-axis indicates the observed $-\log_{10}$ (p-values). Subdivision of the markers on MAF shows that lower minor allele frequencies are shifting from the observed/expected at the start (see light blue line in 1A). The QQ plot with all markers having a MAF >1%, and all NA's excluded, shows the observed shift was caused by including lower allele frequencies (figure 1B). Little inflation was observed for MAF > 20% (1A). A permutation-based approach, of 1000.000 permutations, was investigated for removing the inflation. QQ plot 1Cof the permutation-based approach , showing all markers with a MAF >1%, shows no removal of this inflation.

1A:

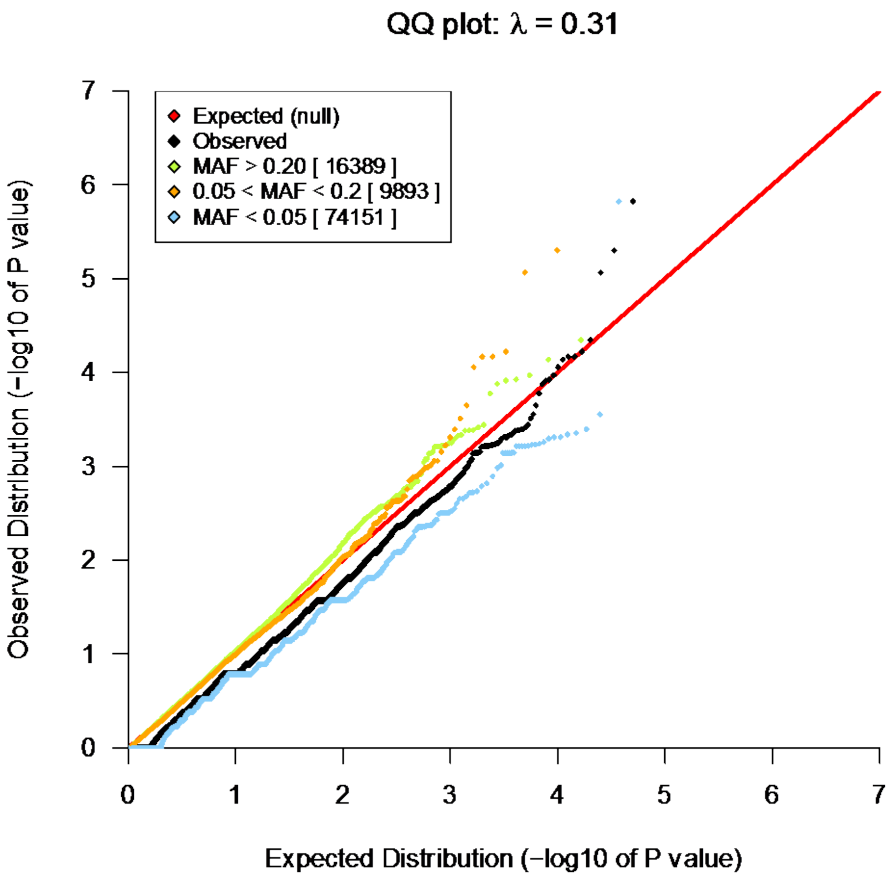

1B:

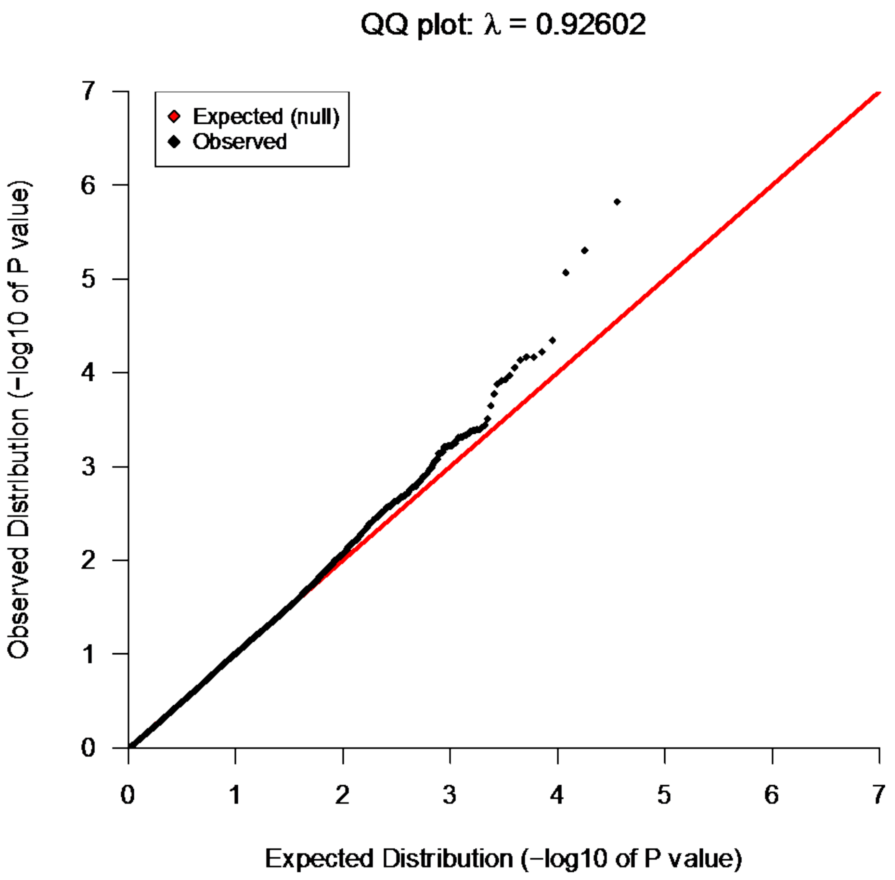
QQ plot: λ = 0.92602
Expected (null)
Observed
Observed Distribution (−log10 of P value)
Expected Distribution (−log10 of P value)
0
1
2
3
4
5
6
7

1C

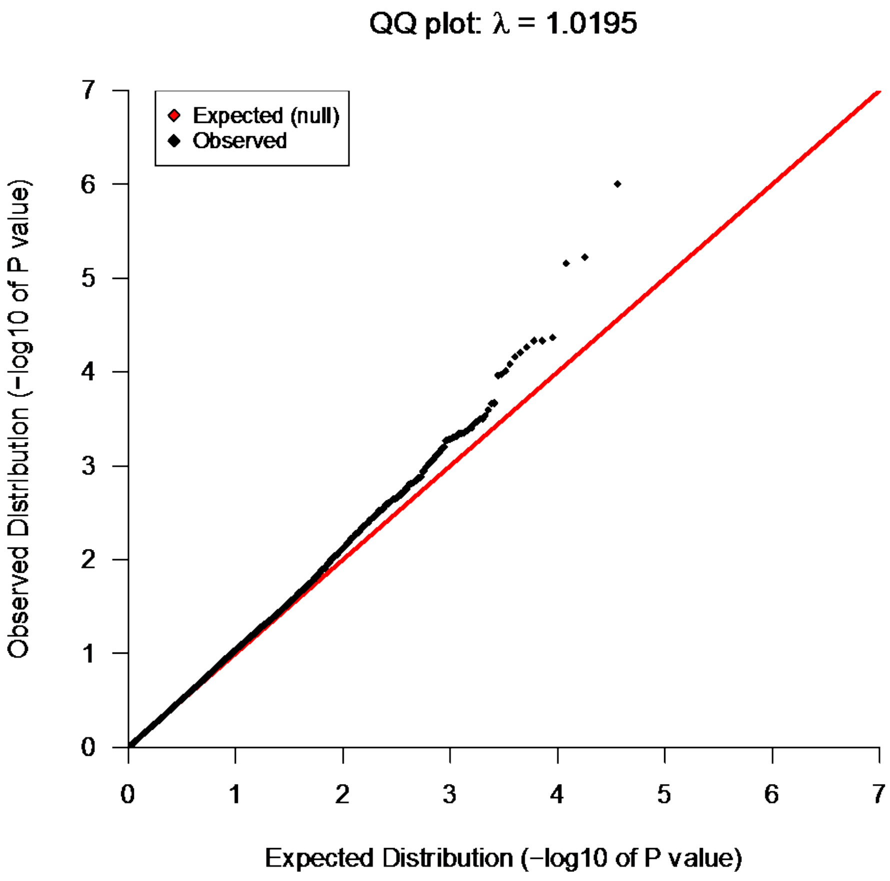
QQ plot: λ = 1.0195
Expected (null)
Observed
Observed Distribution (−log10 of P value)
Expected Distribution (−log10 of P value)
0
1
2
3
4
5
6
7
0
1
2
3
4
5
6
7

Supplementary Table 1 Susceptibility to pneumococcal meningitis (54 lowest P-values; P<0.001 / Fisher exact test)

Chr	Basepair	Rs number	Gene	Alleles		A1 allele frequency		P-value		OR (+/- 95 CI)
				A1	A2	Cases	Control	Unadjusted	BONF	
1	103354135	rs139064549	COL11A1	G	C	0.0393	0.0126	1.51×10^{-6}	0.15	3.21 (2.05 - 5.02)
2	72533963	rs9309464	EXOC6B	G	A	0.146	0.206	6.01×10^{-5}	1	0.66 (0.54 - 0.81)
14	44975606	rs3809429	FSCB	G	A	0.123	0.0784	6.80×10^{-5}	1	1.65 (1.3 - 2.09)
14	44975052	rs3825630	FSCB	A	C	0.123	0.784	6.80×10^{-5}	1	1.65 (1.3 - 2.09)
1	88745673	rs617169	-	G	A	0.281	0.353	7.33×10^{-5}	1	7.18 (6.09 - 8.47)
14	44974966	rs1959379	FSCB	A	G	0.123	0.0785	8.81×10^{-5}	1	1.64 (1.3 - 2.09)
16	71988106	rs9921412	PKD1L3	G	A	0.3305	0.2628	0.000107	1	1.39 (1.18 - 1.63)
9	7174673	rs913588	KDM4C	G	A	0.532	0.4578	0.000119	1	1.35 (1.16 - 1.57)
7	157510195	rs6459804	PTPRN2	A	G	0.4606	0.3878	0.0001221	1	1.35 (1.16 - 1.57)
2	15807637	rs73917150	-	C	A	0.5049	0.4319	0.0001328	1	1.34 (1.15 - 1.56)
11	18267005	rs186939156	SAA2-SAA4	A	G	0.4361	0.5083	0.0001682	1	7.48 (6.43 - 8.71)
5	63889280	rs6556756	LOC101927835	C	A	0.1634	0.115	0.0002247	1	1.50 (1.22 - 1.85)
7	55762157	rs6943029	-	A	G	0.1351	0.09198	0.0003107	1	1.54 (1.23 - 1.94)
1	18201318	rs563835	-	G	A	0.5491	0.4807	0.0003614	1	1.32 (1.13 - 1.53)
22	45312306	rs114948338	PHF21B	A	G	0.03194	0.01327	0.0004023	1	2.45 (1.53 - 3.94)
12	112871372	rs11066301	PTPN11	G	A	0.3673	0.4344	0.0004109	1	0.76 (0.65 - 0.88)

9	4836811	rs7861943	RCL1	A	G	0.2574	0.32	0.0004137	1	7.37 (6.21 - 8.73)
16	71509796	rs8050871	ZNF19	G	C	0.3919	0.3272	0.0004185	1	1.33 (1.14 - 1.55)
4	69227787	rs114673603	DDX60	G	A	0.02703	0.01014	0.0004598	1	2.71 (1.61 - 4.57)
1	20978058	rs650616	DDOST, PINK1. PINK1-AS	G	A	0.3354	0.4008	0.0004651	1	0.75 (0.64 - 0.88)
12	112486818	rs17696736	NAA25	G	A	0.3673	0.4336	0.0004744	1	0.76 (0.65 - 0.89)
2	19563602	rs12993599	STK36	A	G	0.0258	0.05357	0.00049	1	0.47 (0.30 - 0.74)
2	19553468	rs16859180	STK36	A	G	0.0258	0.05357	0.00049	1	0.47 (0.30 - 0.74)
1	75913826	rs1857353	SLC44A5	A	G	0.03686	0.06829	0.0004916	1	0.52 (0.36 - 0.77)
10	48371397	rs79620213	ZNF488	A	G	0.0172	0.004826	0.0005077	1	3.61 (1.82 - 7.17)
1	57805650	rs11537583	CD5L	A	T	0.01843	0.00555	0.0005531	1	3.36 (1.75 - 6.48)
12	113325629	rs4141253	RPH3A	G	A	0.4091	0.3448	0.000566	1	1.32 (1.13 - 1.53)
10	115377224	rs150955833	NRAP	C	A	0.01106	0.001931	0.0005768	1	5.78 (2.22 - 15.03)
4	8229140	rs141562329	SH3TC1	A	C	0.009877	0.001448	0.0005886	1	6.88 (2.38 - 19.88)
1	183918820	rs2378789	COLGALT2	C	A	0.3538	0.4184	0.0005944	1	0.76 (0.65 - 0.89)
8	55914515	rs4236946	-	A	G	0.02948	0.05792	0.0005964	1	0.49 (0.32 - 0.76)
2	233537125	rs11550699	EFHD1	G	A	0.3305	0.3943	0.0006014	1	0.76 (0.65 - 0.89)
1	20474812	rs150487984	PLA2G2F	A	G	0.02948	0.01182	0.0006051	1	2.54 (1.55 - 4.16)
17	1686410	rs9902398	SMYD4	A	G	0.3403	0.2794	0.0006092	1	1.33 (1.13 - 1.56)

2	31178838	rs145021697	GALNT14.	A	G	0.006143	0.0002413	0.000612	1	25.61 (2.99 - 219.50)
6	13604718	rs181494470	SIRT5	G	A	0.006143	0.0002413	0.000612	1	25.61 (2.99 - 219.50)
17	42225445	rs116266545	C17orf53	A	G	0.006143	0.0002413	0.000612	1	25.61 (2.99 - 219.50)
1	146508934	rs12122100	LOC728989	A	G	0.2303	0.2888	0.0006159	1	0.74 (0.62 - 0.88)
11	25929959	rs1441519	LOC105376597	A	C	0.3624	0.4271	0.0006179	1	0.76 (0.65 - 0.89)
11	57381989	rs4926	SERPING1	A	G	0.3071	0.2486	0.0006251	1	1.34 (1.14 - 1.58)
12	112072424	rs11065987	-	G	A	0.36	0.424	0.0007028	1	0.76 (0.65 - 0.89)
2	26537317	rs150700942	ADGRF3	G	A	0.0172	0.005068	0.0007204	1	3.44 (1.74 - 6.79)
8	30702740	rs148862989	TEX15	G	A	0.004914	0	0.0007221	1	NA
10	103342648	rs139871590	POLL	A	G	0.004914	0	0.0007221	1	NA
11	94906505	rs139859559	SESN3	A	G	0.004914	0	0.0007221	1	NA
11	66834221	rs200747179	RHOD	G	A	0.004914	0	0.0007221	1	NA
6	25870542	rs1165205	SLC17A3	T	A	0.5369	0.4715	0.0007292	1	1.30 (1.12 - 1.51)
7	122547130	rs6952251	-	A	C	0.4398	0.5051	0.0007343	1	0.77 (0.66 - 0.89)
8	145661675	rs7830832	TONSL	A	G	0.4189	0.4829	0.0008346	1	0.77 (0.66 - 0.9)
12	111910219	rs10774625	ATXN2	A	G	0.4386	0.5029	0.0008446	1	0.77 (0.66 - 0.9)
7	1097183	rs11761941	C7orf50, GPR146	A	G	0.2101	0.1612	0.0008784	1	1.38 (1.15 - 1.67)
9	35661943	rs72727021	ARHGEF39, CCDC107	C	A	0.1388	0.09821	0.0008832	1	1.48 (1.18 - 1.85)

2	72707874	rs653220	EXOC6B	G	A	0.1794	0.2317	0.0009096	1	0.72 (0.60 - 0.88)
12	64173807	rs61935924	TMEM5	A	G	0.14	0.0999	0.00097	1	1.47 (1.18 - 1.83)

* NA: Not applicable

Supplementary figure 2: Multidimensional scaling analysis (MDS) of the MeninGene and control samples. MDS was used to identify the population stratification and used to select those samples that cluster with the European descent (CEU). Colors used indicate the HapMap populations origin namely, African (red), Asian (purple), New world (green), European (bleu) and MeninGene samples in grey. The dashed lines are the cut off values for European descent.

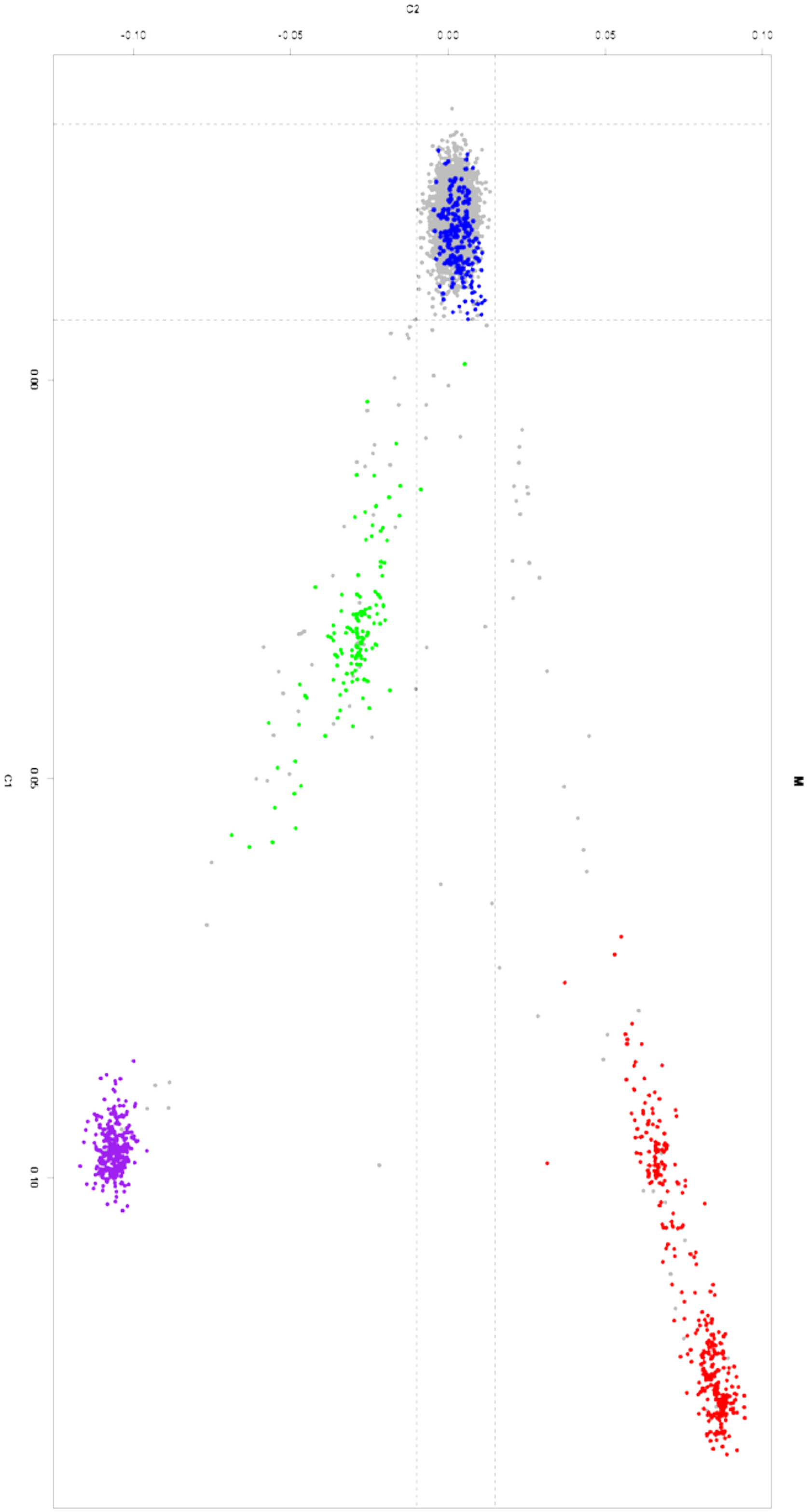

Supplement: Supplementary Information [file srep29351-s1.pdf]
